# Prevalence of Dyslipidemia and Associated Factors Among Adult Outpatients at Jimma Medical Center, Southwest Ethiopia: A Cross‐Sectional Study

**DOI:** 10.1002/hsr2.73370

**Published:** 2026-10-08

**Authors:** Amare Hailu Ashine, Besufekad Kero, Ermias Habte Gebremichael, Melat Dadigeba, Yonas Degelo Geremamo, Esmael Mohamed Dawud, Endalew Dessie Birara, Yonas Damtew Bejiga, Sisay Tagese Tafese, Tamirat Godebo Woyimo

**Affiliations:** ^1^ Department of Internal Medicine Jimma University Jimma Ethiopia; ^2^ Addis Ababa University Addis Ababa city Ethiopia; ^3^ Department of Internal Medicine Addis Ababa University Addis Ababa city Ethiopia; ^4^ Department of Clinical Radiology Addis Ababa University Addis Ababa city Ethiopia

**Keywords:** adult outpatients, associated factors, cardiovascular risk, Dyslipidemia, Ethiopia, Jimma Medical Center, prevalence

## Abstract

**Background and Aims:**

Dyslipidemia is a major modifiable cardiovascular risk factor that significantly contributes to the global burden of atherosclerotic disease. In Ethiopia, particularly among adult outpatients in routine care settings, the magnitude and determinants of lipid abnormalities have not been adequately characterized. This study aimed to assess the prevalence of dyslipidemia and identify associated factors among adult patients attending the outpatient department of Jimma Medical Center, Southwest Ethiopia.

**Methods:**

A facility‐based cross‐sectional study was conducted from January 10 to March 20, 2026, among 376 adult outpatients at Jimma Medical Center. A target sample of 386 participants was determined using prevalence and double population proportion approaches, and consecutive sampling was used; 376 participants ultimately participated, yielding a 97.4% response rate. Data were collected using a structured and pre‐tested questionnaire and data extraction checklist. Dyslipidemia was defined according to the National Cholesterol Education Program Adult Treatment Panel III (NCEP ATP III) criteria. Bivariable and multivariable binary logistic regression analyses were performed to identify factors independently associated with dyslipidemia. Variables with *p*‐value ≤ 0.25 in the bivariable analysis were entered into the multivariable model, and adjusted odds ratios with 95% confidence intervals were reported. Statistical significance was declared at *p*‐value < 0.05.

**Results:**

A total of 376 participants were enrolled, with a mean age of 41.5 ± 13.6 years, and 52.1% were male. The overall prevalence of dyslipidemia was 60.4% (95% CI: 55.4–65.3%) among untreated adult outpatients included in the study. In the multivariable logistic regression analysis, age category 50–64 years (adjusted odds ratio [AOR] = 2.32, 95% CI: 1.20–4.49), obesity (AOR = 4.24, 95% CI: 2.34–7.66), and presence of hypertension (AOR = 1.81, 95% CI: 1.14–2.90) were independently associated with increased odds of dyslipidemia. Conversely, being a farmer was significantly associated with lower odds of dyslipidemia (AOR = 0.38, 95% CI: 0.15–0.96).

**Conclusions:**

The prevalence of dyslipidemia among adult outpatients at Jimma Medical Center was high, affecting more than half of the study participants. Older age, obesity, and hypertension were significant predictors of dyslipidemia, while farming occupation appeared protective. Integrating routine lipid screening into outpatient services and implementing targeted weight management and hypertension control programs may help reduce the cardiovascular risk burden in this population.

**Abbreviations**
AOR: adjusted odds ratio
ART: antiretroviral therapy
BMI: body mass index
CHD: coronary heart disease
CI: confidence interval
CKD: chronic kidney disease
COR: Crude Odds Ratio
DALYs: disability‐adjusted life years
DBP: diastolic blood pressure
DEGS1: German Health Interview and Examination Survey for Adults
DM: diabetes mellitus
FBS: fasting blood sugar
GBD: Global Burden of Disease
HDL‐C: high‐density lipoprotein cholesterol
IHD: ischemic heart disease
LDL‐C: low‐density lipoprotein cholesterol
NHANES: National Health and Nutrition Examination Survey
T2DM: type 2 diabetes mellitus
TC: total cholesterol
TG: triglycerides
TIA: transient ischemic attack

## 1 Background

Dyslipidemia is a metabolic disorder characterized by abnormalities in serum lipid levels, including elevated total cholesterol, low‐density lipoprotein cholesterol (LDL‐C), triglycerides, and/or reduced high‐density lipoprotein cholesterol (HDL‐C) [1, 2]. It is one of the major modifiable risk factors for atherosclerotic cardiovascular disease and contributes substantially to the growing global burden of non‐communicable diseases. Persistent lipid abnormalities accelerate endothelial dysfunction, atherosclerotic plaque formation, and vascular inflammation, thereby increasing the risk of ischemic heart disease, stroke, and peripheral vascular disease [2, 3, 4].

Globally, dyslipidemia has become a significant public health concern, particularly in low‐ and middle‐income countries undergoing rapid epidemiological transition. Recent evidence indicates that millions of deaths and disability‐adjusted life years are attributable to elevated LDL‐C and other lipid abnormalities each year [5, 6]. In Africa, the burden of dyslipidemia is increasing due to urbanization, sedentary lifestyles, unhealthy dietary patterns, obesity, and the rising prevalence of hypertension and diabetes mellitus. Ethiopia is similarly experiencing a growing burden of cardio‐metabolic disorders, with previous studies reporting a high prevalence of dyslipidemia among different adult populations [6, 7].

Several studies have identified older age, obesity, hypertension, diabetes mellitus, physical inactivity, smoking, and unhealthy lifestyle behaviors as important predictors of dyslipidemia. These risk factors frequently coexist and synergistically increase cardiovascular risk [8, 9, 10]. Despite the increasing burden of dyslipidemia in Ethiopia, most available studies have focused on selected populations such as diabetic or hypertensive patients, while evidence among general adult outpatient populations remains limited [6].

Previous Ethiopian studies have predominantly examined dyslipidemia in community‐based populations, occupational groups, or patients with specific chronic conditions such as diabetes and hypertension. In contrast, the present study focuses on a heterogeneous population of adults receiving routine outpatient care, including patients with different clinical conditions and cardiovascular risk profiles. This setting is clinically relevant because outpatient services represent an important opportunity for identifying previously unrecognized lipid abnormalities and integrating lipid assessment with management of other cardiovascular risk factors. Therefore, the present study provides facility‐based evidence on the burden and determinants of dyslipidemia in a real‐world outpatient population at a major referral hospital in southwestern Ethiopia. Furthermore, routine lipid screening is not consistently practiced in many healthcare settings, leading to delayed diagnosis and management of lipid abnormalities. Understanding the magnitude of dyslipidemia and its associated factors among adult outpatients is essential for strengthening early screening, prevention, and integrated cardiovascular risk management strategies. Therefore, this study aimed to determine the prevalence of dyslipidemia and identify factors associated with dyslipidemia among adult outpatients attending Jimma Medical Center, Southwest Ethiopia, thereby providing evidence to support integrated lipid screening and cardiovascular risk assessment in outpatient care.

## 2 Methods

### 2.1 Study Design and Setting

A facility‐based cross‐sectional study was conducted from January 10 to March 20, 2026, at the outpatient department of Jimma Medical Center, Southwest Ethiopia. Jimma Medical Center is the largest teaching and referral hospital in the southwestern part of the country, serving a catchment population of more than 15 million people from Oromia, Southwest Ethiopia Peoples’ Region, and Gambella People's Regional State. The outpatient department provides general medical care for a diverse population of adult patients with acute and chronic health problems.

### 2.2 Study Population and Eligibility Criteria

Participants were eligible if they were adults aged ≥ 18 years who attended the outpatient department of Jimma Medical Center during the study period and had available information on lipid profile and relevant clinical and sociodemographic characteristics. Patients were excluded if they had incomplete information required for the analysis or were unable to provide the required information. Patients receiving lipid‐lowering medication were excluded from the study. Patients who were currently receiving lipid‐lowering therapy were excluded because lipid‐lowering treatment may modify measured lipid concentrations and thereby complicate the assessment of untreated dyslipidemia using lipid concentration‐based diagnostic thresholds.

Eligibility was determined before assessment and classification of the dyslipidemia outcome. Participants who were receiving lipid‐lowering medication at the time of recruitment were excluded from the study and therefore were not included in the analysis of dyslipidemia prevalence or associated factors. Consequently, all participants included in the final analysis were assessed using their measured lipid concentrations rather than treatment status.

### 2.3 Sample Size

The sample size was determined using both a single population proportion formula for estimating the prevalence of dyslipidemia and a double population proportion approach for assessing factors associated with dyslipidemia, using Epi Info StatCalc version 7.2.2.6. For the prevalence estimate, a previously reported dyslipidemia prevalence of 66.7% from a community‐based study in Mekelle, Ethiopia, a 5% margin of error, and a 95% confidence level yielded a minimum sample size of 349 participants. After allowing for a 10% anticipated non‐response rate, the target sample size was 386. The double population proportion calculations for the selected associated factors yielded sample sizes smaller than 386; therefore, 386 was retained as the planned sample size. Of the 386 participants targeted, 376 participated in the study, giving a response rate of 97.4%, and these 376 participants constituted the final analytic sample. The difference between the planned and achieved sample size was due to non‐participation of 10 eligible individuals.

Although the sample size calculation included a double population proportion approach for selected associated factors, the study was not separately powered to detect associations for every individual covariate considered in the multivariable model. The final analysis included 376 participants, of whom 227 had dyslipidemia, providing a substantial number of outcome events for the fitted logistic regression model. Nevertheless, the precision and ability to detect associations may have varied across individual predictors, particularly for variables with fewer observations or smaller effect sizes.

### 2.4 Sampling Technique

A consecutive sampling technique was employed to recruit study participants. Every eligible adult patient who presented to the outpatient department during the data collection period and met the inclusion criteria was invited to participate until the required sample size was achieved.

### 2.5 Data Collection Procedures and Measurements

Data were collected using a structured, pretested interviewer‐administered questionnaire and a data extraction checklist. The questionnaire captured socio‐demographic characteristics (age, sex, residence, education, occupation), behavioral and lifestyle factors (alcohol use, cigarette smoking, khat chewing, physical activity), and medical history. Physical measurements, including weight, height, and waist circumference, were taken using standardized procedures. Body mass index (BMI) was calculated as weight in kilograms divided by height in meters squared (kg/m^2^). Blood pressure was measured on the right arm in a seated position after at least 5 min of rest using a calibrated digital sphygmomanometer.

Fasting blood glucose and lipid profile, including total cholesterol, triglycerides, LDL‐C, and HDL‐C, were obtained through routine clinical laboratory testing. All blood samples were analyzed in the same clinical laboratory using an automated clinical chemistry analyzer, the Cobas 6000 analyzer (c501 module; Roche Diagnostics). LDL‐C was measured directly using an enzymatic reagent‐based method. The same laboratory and analytical platform were used for all lipid measurements to ensure consistency of laboratory assessment across participants. Dyslipidemia was defined according to the National Cholesterol Education Program Adult Treatment Panel III (NCEP ATP III) criteria as the presence of at least one of the following: total cholesterol ≥ 200 mg/dL, triglycerides ≥ 150 mg/dL, LDL‐C ≥ 130 mg/dL, or HDL‐C < 40 mg/dL in men and < 50 mg/dL in women.

### 2.6 Data Quality Assurance

To ensure data quality, the questionnaire was pretested on 5% of the calculated sample at a similar outpatient setting, and necessary modifications were made. Data collectors and supervisors received intensive training on the study objectives, data collection procedures, ethical considerations, and the use of KoboToolbox for electronic data capture. Supervision was conducted daily, and the KoboCollect platform was configured with mandatory fields and skip patterns to minimize missing data and entry errors. After data collection, all records were exported and thoroughly cleaned, including range checks and verification of inconsistencies before analysis.

### 2.7 Data Processing and Statistical Analysis

Data were collected electronically using KoboToolbox on smartphones, exported to Microsoft Excel for cleaning, and then transferred to STATA version 17 for analysis. Descriptive statistics were used to summarize participant characteristics, including frequencies and percentages for categorical variables and mean with standard deviation for continuous variables.

#### 2.7.1 Model‐Building Strategy

Bivariable binary logistic regression was first performed to screen each independent variable for its association with dyslipidemia. Variables with a *p*‐value ≤ 0.25 in the bivariable analysis were considered candidates for the multivariable model. This liberal cut‐off was chosen based on the recommendation of Hosmer and Lemeshow to avoid excluding potentially important confounders that may become statistically significant after adjustment for other variables, while also reducing the risk of residual confounding. A backward stepwise elimination approach was used for multivariable model construction. This method was selected because it allows all candidate variables to be entered simultaneously, evaluates the contribution of each variable while adjusting for all others, and sequentially removes the least significant variable until all remaining variables are statistically significant at *p* < 0.05. Adjusted odds ratios (AOR) with 95% confidence intervals (CI) were calculated for the final model. Variables were selected as candidates for the multivariable model based on three criteria: (1) statistical significance at *p* ≤ 0.25 in bivariable analysis, (2) biological plausibility and clinical relevance as established in the literature (e.g., age, obesity, hypertension, diabetes, CKD), and (3) availability and completeness of data in the study population.

#### 2.7.2 Model Diagnostics

Multicollinearity among the independent variables in the final model was assessed using the variance inflation factor (VIF), with a threshold of 10 indicating problematic multicollinearity. The VIF values for all variables in the final model ranged from 1.04 to 1.38, all below the threshold of 10, indicating no significant multicollinearity. Model fitness was evaluated using the Hosmer–Lemeshow goodness‐of‐fit test. The final model demonstrated adequate fit, with a Hosmer–Lemeshow chi‐square statistic of 7.82 and a non‐significant p‐value of 0.451, indicating that the model adequately describes the data. Statistical significance was declared at *p* < 0.05.

All statistical analyses were performed using Stata version 17 (StataCorp LLC, College Station, TX, USA). Categorical variables were summarized using frequencies and percentages, while continuous variables were summarized using means and standard deviations where appropriate based on their distribution. Bivariable binary logistic regression was used to assess crude associations between independent variables and dyslipidemia, and variables meeting the predefined screening criterion of *p* ≤ 0.25, together with clinically relevant covariates, were considered for multivariable binary logistic regression. Adjusted odds ratios (AORs) with 95% confidence intervals (CIs) were reported to quantify the magnitude and precision of associations. Multicollinearity was assessed using the variance inflation factor, and model fit was evaluated using the Hosmer–Lemeshow goodness‐of‐fit test. Statistical significance was assessed using two‐sided tests with an a priori significance level of α = 0.05. No subgroup analyses were performed.

### 2.8 Operational Definitions

#### 2.8.1 Dyslipidemia

Was defined as the presence of at least one of the following: total cholesterol ≥ 200 mg/dL, triglycerides ≥ 150 mg/dL, LDL‐C ≥ 130 mg/dL, HDL‐C < 40 mg/dL in men or < 50 mg/dL in women [11]. Current use of lipid‐lowering medication was not included as a diagnostic criterion because participants receiving lipid‐lowering therapy were excluded before outcome assessment.

#### 2.8.2 Current Smoker

Participant who has smoked any tobacco product in the last 30 days [12].

#### 2.8.3 Current Alcohol Use

Any consumption of alcoholic beverages in the last 30 days [12].

#### 2.8.4 Insufficient Physical Activity

Engaging in < 150 min of moderate‐to‐vigorous physical activity per week [12].

## 3 Results

### 3.1 Socio‐Demographic Characteristics

A total of 386 eligible participants were targeted for enrollment, of whom 376 participated in the study, yielding a response rate of 97.4%. The 376 participants who completed the study constituted the final analytic sample. The mean age of the participants was 41.5 (± 13.6 SD) years, with the largest proportion of respondents, 180 (47.9%), falling within the middle‐aged category of 18–34 years. Regarding the sex distribution, nearly half, 196 (52.1%) of the participants were males. More than half of the respondents, 217 (57.7%), were urban residents. In terms of educational status, more than one‐third of the participants, 128 (34.0%), attended secondary education (grade 9–12), and the majority of the participants, 220 (58.5%), were married (Table 1).

**Table 1:** Sociodemographic characteristics of patients attending the outpatient department of Jimma Medical Center, Southwest Ethiopia, 2026 (*n* = 376).

Variable	Category	Number (%)
Sex	Male	196 (52.1)
	Female	180 (47.9)
Age	Young Adults (18–34)	180 (47.9)
	Middle‐aged Adults (35–49)	113 (30.1)
	Older Adults (50–64)	65 (17.3)
	Elderly (≥ 65)	18 (4.8)
Educational status	No formal education	60 (16.0)
	Primary (1–8)	121 (32.2)
	Secondary (9–12)	128 (34.0)
	Diploma and above	67 (17.8)
Residence location	Urban	217 (57.7)
	Rural	159 (42.3)
Ethnicity	Oromo	187 (49.7)
	Amhara	73 (19.4)
	Dawuro	41 (10.9)
	Yem	38 (10.1)
	Others*	37 (9.8)
Occupation status	Unemployed	77 (20.5)
	Government employee	82 (21.8)
	Self‐employed	123 (32.7)
	Student	61 (16.2)
	Farmers	33 (8.8)
Religion	Muslim	157 (41.8)
	Orthodox	107 (28.5)
	Protestant	81 (21.5)
	Catholic	15 (4.0)
	Others**	16 (4.3)

* Includes Kefficho, Gurage, and Tigray.

** Includes traditional beliefs and Wakeffata.

### 3.2 Clinical and Metabolic Characteristics

The analysis of clinical and metabolic characteristics revealed significant metabolic concerns. Only 121 (32.2%) of participants maintained a normal Body Mass Index (BMI). Regarding the lipid profile, abnormal fractions were highly prevalent: 315/376 (83.8%) of participants had elevated LDL‐C (≥ 130 mg/dL), 215/376 (57.2%) had high total cholesterol (≥ 200 mg/dL), and 209/376 (55.6%) had high triglycerides (≥ 150 mg/dL). Additionally, 126/376 (33.5%) had low HDL‐C levels (Table 2).

**Table 2:** Clinical and metabolic characteristics of study participants (*n* = 376).

Variable	Category	Number (%)
BMI category	Normal	121 (32.2%)
	Overweight	133 (35.4%)
	Obese	122 (32.5%)
Total cholesterol	High (≥ 200 mg/dL)	215 (57.2%)
	Normal (< 200 mg/dL)	161 (42.8%)
Triglycerides	High (≥ 150 mg/dL)	209 (55.6%)
	Normal (< 150 mg/dL)	167 (44.4%)
LDL cholesterol	High (≥ 130 mg/dL)	315 (83.8%)
	Normal (< 130 mg/dL)	61 (16.2%)
HDL cholesterol	Low (≤ 40 mg/dL)	126 (33.5%)
	Normal (> 40 mg/dL)	250 (66.5%)

### 3.3 Behavioral and Lifestyle Characteristics

Approximately one‐quarter of the participants, 85 (22.6%), reported a lifetime history of cigarette smoking, while a smaller minority, 24 (6.4%), had ever used shisha. Regarding other substance use, more than half of the respondents, 207 (55.1%), reported khat chewing, and 133 (35.4%) had used alcohol in their lifetime. Regarding physical activity, more than one‐third of the participants, 134 (35.6%), reported no engagement in vigorous physical activity. In contrast, 116 (30.9%) participants maintained a high frequency of moderate‐intensity activity, engaging five or more times per week (Table 3).

**Table 3:** Behavioral risk factors and lifestyle characteristics of patients attending the outpatient department of Jimma Medical Center, Southwest Ethiopia, 2026 (*n* = 376).

Variable	Category	Number (%)
Alcohol drinker	Yes	133 (35.4)
	No	243 (64.6)
Cigarette smoker	Yes	85 (22.6)
	No	291 (77.4)
Khat chewer	Yes	207 (55.1)
	No	169 (44.9)
Vigorous physical activity	≥ 3 times/week	122 (32.5)
	1–2 times/week	120 (31.9)
	None	134 (35.6)
Moderate physical activity	≥ 5 times/week	116 (30.9)
	3–4 times/week	94 (25.0)
	1–2 times/week	102 (27.1)
	None	64 (17.0)

### 3.4 Comorbidities

More than one‐third of the participants, 146 (38.8%), had been diagnosed with Diabetes Mellitus, while one‐third, 126 (33.5%), were hypertensive. Regarding renal and cardiovascular health, nearly one‐in‐six participants, 61 (16.2%), had Chronic Kidney Disease (CKD), and 46 (12.2%) were living with Ischemic Heart Disease (IHD). The result also showed that a smaller proportion of the study population had experienced severe cardiovascular events or complications, with 20 (5.3%) having Heart Failure and 17 (4.5%) having a history of Stroke or Transient Ischemic Attack (TIA) (Figure 1).

**Figure 1:**
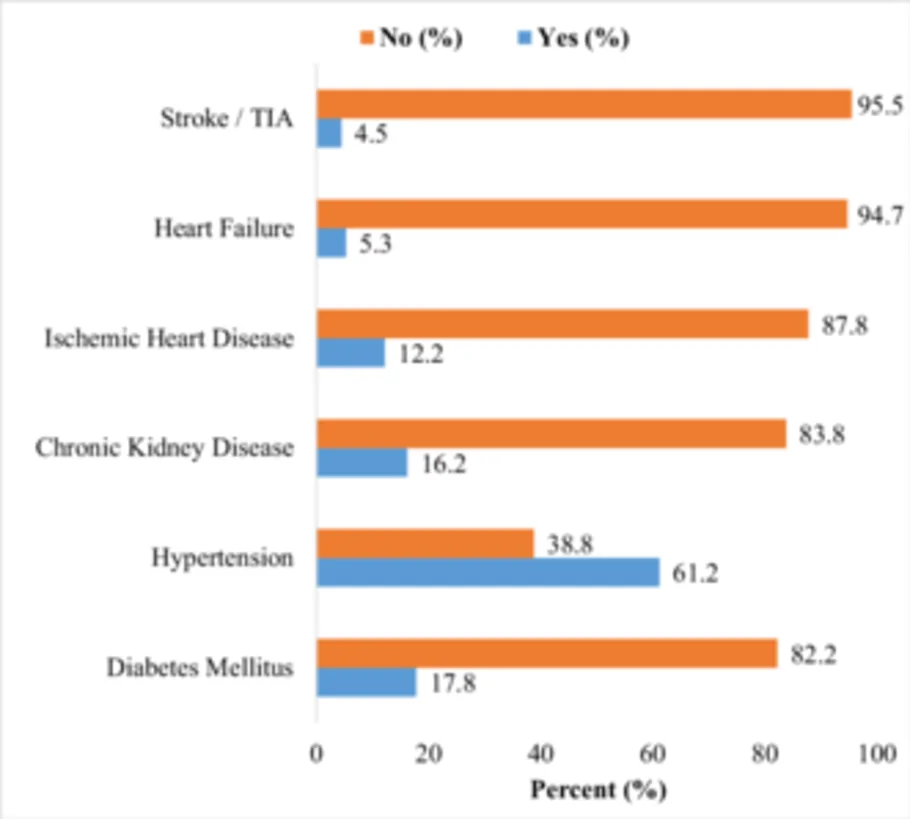
Clinical and comorbidity profile of patients attending the outpatient department of Jimma Medical Center, Southwest Ethiopia, 2026 (*n* = 376).

### 3.5 Prevalence of Dyslipidemia Among Adult Outpatients at Jimma Medical Center

The overall prevalence of dyslipidemia among patients attending the outpatient department of Jimma Medical Center was 227 (60.4%) [95% CI: 55.4%–65.3%] among untreated adult outpatients included in the study (Figure 2).

**Figure 2:**
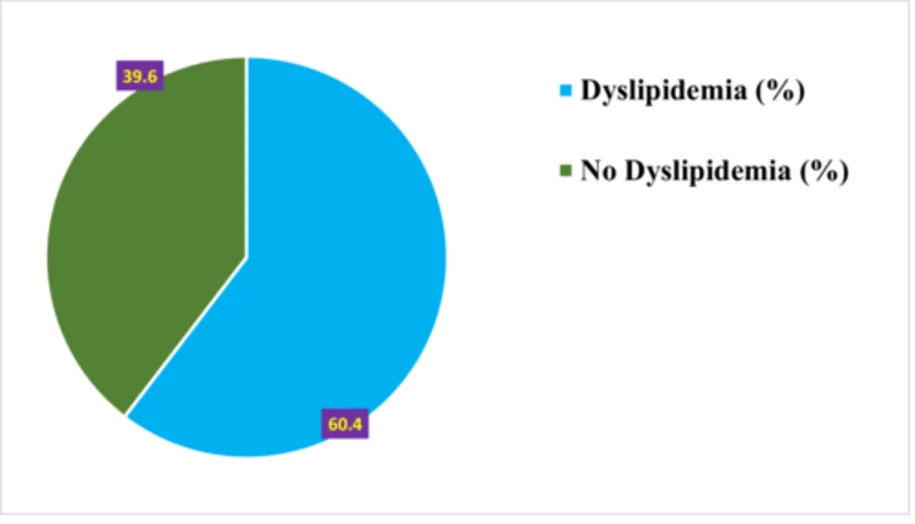
Prevalence of dyslipidemia among patients attending the outpatient department of Jimma Medical Center, Southwest Ethiopia, 2026 (*n* = 376).

### 3.6 Factors Associated With Dyslipidemia Among Patients Attending the Outpatient Department of Jimma Medical Center

In the bivariable logistic regression analysis, several covariates were evaluated to determine their association with dyslipidemia. Variables were considered candidates for the multivariable model if they met the predetermined cut‐off of *p* ≤ 0.25. The analysis identified that age category, occupation, vigorous physical activity, hypertension status, chronic kidney disease (CKD), body mass index (BMI) category, and diabetes mellitus (DM) were significantly associated with dyslipidemia and served as candidates for the multivariable analysis (Table 4).

**Table 4:** Bivariable logistic regression analysis of candidate variables for the multivariable analysis of dyslipidemia among adult outpatients at Jimma Medical Center, Southwest Ethiopia, 2026 (*n* = 376).

Variable	Category	Number (%)	Dyslipidemia		COR (95% CI)	*p* value
			Yes (%)	No (%)		
Age category	18–34 years	180 (47.9)	98 (54.4)	82 (45.6)	1	
	35–49 years	113 (30.1)	69 (61.1)	44 (38.9)	1.312 (0.813–2.117)	0.266
	50–64 years	65 (17.3)	47 (72.3)	18 (27.7)	2.185 (1.178–4.051)	**0.013** *
	≥ 65 years	18 (4.8)	5 (27.8)	5 (27.8)	2.176 (0.745–6.357)	0.155
Sex	Male	196 (52.1)	120 (61.2)	76 (38.8)	1	
	Female	180 (47.9)	107 (59.4)	73 (40.6)	0.928 (0.614–1.404)	0.724
Educational level	No formal education	60 (16.0)	36 (60.0)	24 (40.0)	1	
	Primary (1–8)	121 (32.2)	79 (65.3)	42 (34.7)	1.254 (0.663–2.373)	0.487
	Secondary (9–12)	128 (34.0)	75 (58.6)	53 (41.4)	0.943 (0.505–1.762)	0.855
	Diploma and above	67 (17.8)	37 (55.2)	30 (44.8)	0.822 (0.406–1.666)	0.587
Occupation status	Unemployed	77 (20.5)	45 (58.4)	32 (41.6)	1	
	Government employee	82 (21.8)	54 (65.9)	28 (34.1)	1.371 (0.721–2.609)	0.336
	Self‐employed	123 (32.7)	80 (65.0)	43 (35.0)	1.323 (0.737–2.376)	0.349
	Student	61 (16.2)	36 (59.0)	25 (41.0)	1.024 (0.517–2.026)	0.946
	Farmers	33 (8.8)	12 (36.4)	21 (63.6)	0.406 (0.175–0.943)	**0.036** *
Residence	Urban	217 (57.7)	135 (62.2)	82 (37.8)	1.199 (0.790–1.820)	0.394
	Rural	159 (42.3)	92 (57.9)	67 (42.1)	1	
BMI Category	Normal	121 (32.2)	58 (47.9)	63 (52.1)	1	
	Overweight	133 (35.4%)	74 (55.6)	59 (44.4)	1.362 (0.831–2.233)	0.220
	Obese	122 (32.5%)	95 (77.9)	27 (22.1)	3.822 (2.190–6.669)	0.000**
Alcohol drinker	Yes	133 (35.4)	148 (60.9)	95 (39.1)	1	
	No	243 (64.6)	79 (59.4)	54 (40.6)	0.939 (0.610–1.446)	0.775
Cigarette	Yes	85 (22.6)	46 (54.1)	39 (45.9)	0.717 (0.440–1.168)	**0.181** *
	No	291 (77.4)	181 (62.2)	110 (37.8)	1	
Khat chewer	Yes	207 (55.1)	123 (59.4)	84 (40.6)	1	
	No	169 (44.9)	104 (61.5)	65 (38.5)	1.093 (0.721–1.656)	0.676
Moderate physical activity	≥ 3 times/week	122 (32.5)	77 (63.1)	45 (36.9)	1	
	1–2 times/week	120 (31.9)	68 (56.7)	52 (43.3)	0.764 (0.456–1.280)	0.307
	None	134 (35.6)	82 (61.2)	52 (38.8)	0.922 (0.556–1.528)	0.752
Vigorous physical activity	≥ 5 times/week	116 (30.9)	63 (54.3)	53 (45.7)	1	
	3–4 times/week	94 (25.0)	61 (64.9)	33 (35.1)	1.555 (0.889–2.721)	0.122
	1–2 times/week	102 (27.1)	57 (55.9)	45 (44.1)	1.066 (0.624–1.819)	0.816
	None	64 (17.0)	46 (71.9)	18 (28.1)	2.150 (1.116–4.143)	**0.022** *
Hypertension	Yes	230 (61.2)	149 (64.8)	81 (35.2)	1.604 (1.051–2.448)	0.029*
	No	146 (38.8)	78 (53.4)	68 (46.6)	1	
Diabetes	Yes	67 (17.8)	49 (73.1)	18 (26.9)	2.003 (1.116–3.598)	**0.020** *
	No	309 (82.2)	178 (57.6)	131 (42.4)	1	
CKD	Yes	61 (16.2)	27 (44.3)	34 (55.7)	0.652 (0.378–1.123)	0.123
	No	315 (83.8)	200 (63.5)	115 (36.5)	1	

* Significant at *p*‐value ≤ 0.25 1 = references.

In the multivariable logistic regression analysis, the covariates age category, occupation status, BMI category, hypertension, and chronic kidney disease were found to be the independent predictors of dyslipidemia among the study participants.

Accordingly, participants in the age group of 50–64 years were more than twice as likely to develop dyslipidemia compared to those in the reference age group of 18–34 years (AOR = 2.32, 95% CI: 1.20–4.49). Occupation status was also an independent predictor; specifically, farmers had 62% lower odds of having dyslipidemia compared to the reference group of unemployed individuals (AOR = 0.38, 95% CI: 0.15–0.96).

BMI category remained a highly significant independent predictor in the final model. Participants who were obese were four times more likely to have dyslipidemia than those with a normal BMI (AOR = 4.24, 95% CI: 2.34–7.66). Also, the presence of hypertension significantly predicted the occurrence of dyslipidemia. To this fact, hypertensive participants were 81% more likely to have dyslipidemia as compared to those without hypertension (AOR = 1.81, 95% CI: 1.14–2.90) (Table 5).

**Table 5:** Multivariable logistic regression analysis of factors associated with dyslipidemia among adult outpatients at Jimma Medical Center, Southwest Ethiopia, 2026 (*n* = 376).

Variable	Category	Number (%)	Dyslipidemia		AOR (95% CI)	*p* value
			Yes (%)	No (%)		
Age category	18–34 years	180 (47.9)	98 (54.4)	82 (45.6)	1	
	35–49 years	113 (30.1)	69 (61.1)	44 (38.9)	1.254 (0.739–2.128)	0.402
	50–64 years	65 (17.3)	47 (72.3)	18 (27.7)	2.324 (1.203–4.490)	**0.012** *
	≥ 65 years	18 (4.8)	5 (27.8)	5 (27.8)	2.962 (0.935–9.382)	0.065
Occupation status	Unemployed	77 (20.5)	45 (58.4)	32 (41.6)	1	
	Gov't employee	82 (21.8)	54 (65.9)	28 (34.1)	1.371 (0.676–2.779)	0.382
	Self‐employed	123 (32.7)	80 (65.0)	43 (35.0)	1.456 (0.768–2.760)	0.250
	Student	61 (16.2)	36 (59.0)	25 (41.0)	1.062 (0.506–2.231)	0.873
	Farmers	33 (8.8)	12 (36.4)	21 (63.6)	0.381 (0.151–0.960)	**0.041** *
BMI Category	Normal	121 (32.2)	58 (47.9)	63 (52.1)	1	
	Overweight	133 (35.4%)	74 (55.6)	59 (44.4)	1.381 (0.816–2.338)	0.229
	Obese	122 (32.5%)	95 (77.9)	27 (22.1)	4.236 (2.342–7.662)	**0.000** **
Cigarette	Yes	85 (22.6)	46 (54.1)	39 (45.9)	1	
	No	291 (77.4)	181 (62.2)	110 (37.8)	0.652 (0.378–1.123)	0.123
Vigorous physical activity	≥ 5 times/week	116 (30.9)	63 (54.3)	53 (45.7)	1	
	3–4 times/week	94 (25.0)	61 (64.9)	33 (35.1)	0.725 (0.410–1.282)	0.269
	1–2 times/week	102 (27.1)	57 (55.9)	45 (44.1)	1.066 (0.624–1.819)	0.816
	None	64 (17.0)	46 (71.9)	18 (28.1)	0.836 (0.478–1.462)	0.530
Hypertension	Yes	230 (61.2)	149 (64.8)	81 (35.2)	1.814 (1.136–2.895)	**0.013** *
	No	146 (38.8)	78 (53.4)	68 (46.6)	1	
Diabetes	Yes	67 (17.8)	49 (73.1)	18 (26.9)	0.923 (0.580–1.470)	0.737
	No	309 (82.2)	178 (57.6)	131 (42.4)	1	
CKD	Yes	61 (16.2)	27 (44.3)	34 (55.7)	0.652 (0.378–1.123)	0.123
	No	315 (83.8)	200 (63.5)	115 (36.5)	1	

* Significant at *p*‐value ≤ 0.25.

** Significant at *p*‐value ≤ 0.25 1 = references.

#### 3.6.1 Model Diagnostics

The final multivariable logistic regression model showed adequate goodness‐of‐fit (Hosmer–Lemeshow *χ* ^2^ = 7.82, df = 8, *p* = 0.451). Multicollinearity was not a concern, with VIF values for all variables in the final model ranging from 1.04 to 1.38, all below the threshold of 10 (mean VIF = 1.19). The model correctly classified 71.5% of cases.

## 4 Discussion

This study assessed the prevalence of dyslipidemia and its associated factors among adult outpatients at Jimma Medical Center. More than half of the study participants, 60.4% (95% CI: 55.4%–65.3%), had dyslipidemia. However, because patients receiving lipid‐lowering therapy were excluded, this estimate represents untreated adult outpatients and may underestimate the underlying burden of dyslipidemia in the broader outpatient population. The study also revealed that age category, occupation status, BMI category, and hypertension were significantly associated with dyslipidemia among the study participants.

The finding from this study, which revealed a dyslipidemia prevalence of 60.4%, is in line with the previous Ethiopian systematic review and meta‐analysis, which estimated that between 56.6% and 64% of adults in the country meet the criteria for dyslipidemia [5]. It is also consistent with community‐based data from Addis Ababa (63.5%) and Mekelle city (66.7%), which showed a similarly high burden of at least one lipid abnormality [8, 10]. Furthermore, this finding is comparable to studies conducted in other African nations, such as Kenya (68%) and South Africa (67%), where high rates of lipid derangements were documented among adult populations [13, 14].

However, the prevalence in this study is notably higher than the pooled global prevalence values of 28.8% for hypertriglyceridemia and 24.1% for hypercholesterolemia [3]. It is also significantly higher than the reported prevalence in France (23.3%) and the United Kingdom (22.7%) [15, 16]. The possible reason for this variation might be due to the dissimilarity in the study populations and settings. While many of the European studies were based on the general adult population or primary care cohorts, the current study was conducted among hospital outpatients, a group more likely to have underlying comorbidities such as hypertension and diabetes, which are known to be strongly associated with dyslipidemia. The variation might also be attributed to differences in dietary habits, lifestyles, and the increasing trend of obesity in urbanizing Ethiopian centers like Jimma, compared to the established management protocols in high‐income countries.

The proportion of participants with elevated LDL‐C was particularly high, with 83.8% meeting the NCEP ATP III threshold of ≥ 130 mg/dL. Several factors may have contributed to this finding. First, the study population consisted of adult outpatients attending a tertiary referral hospital, who may have had a greater burden of cardiometabolic conditions and other cardiovascular risk factors than the general community population. This referral pattern may have increased the likelihood of observing lipid abnormalities and may partly explain the high LDL‐C prevalence. Second, differences in population characteristics, including the relatively high frequency of hypertension, diabetes mellitus, overweight/obesity, and other comorbidities in this outpatient population, may have contributed to the observed lipid profile. Third, all lipid measurements were performed through routine clinical laboratory testing in the same laboratory using the Cobas 6000 automated clinical chemistry analyzer (c501 module), with LDL‐C measured directly using an enzymatic method. Thus, the high prevalence is unlikely to be attributable to variation in laboratory platforms across study participants, although differences in pre‐analytical factors and the clinical characteristics of the referred outpatient population may still have influenced the observed estimate. The finding should therefore be interpreted in the context of the study population and setting rather than assumed to represent the prevalence of elevated LDL‐C in the general Ethiopian adult population.

The findings of the present study should also be considered within the context of recent advances in the understanding and management of cardiometabolic risk. Dyslipidemia remains an important component of residual cardiovascular risk, and contemporary evidence continues to emphasize the clinical importance of achieving adequate control of atherogenic lipid fractions, particularly LDL‐C. A recent GRADE‐assessed meta‐analysis of randomized trials found that obicetrapib, a cholesteryl ester transfer protein inhibitor, produced substantial reductions in LDL‐C and non‐HDL‐C and improved attainment of recommended LDL‐C targets among adults with dyslipidemia, with or without cardiovascular risk [17]. These findings reinforce the clinical importance of identifying elevated LDL‐C in routine outpatient populations such as that examined in the present study. Similarly, a recent updated meta‐analysis of randomized trials evaluating olezarsen demonstrated substantial reductions in triglyceride and apolipoprotein C‐III levels, with favorable effects on non‐HDL‐C, highlighting the continuing development of therapeutic strategies targeting atherogenic lipid abnormalities and residual cardiometabolic risk [18]. Taken together, these contemporary data support the clinical relevance of detecting and addressing dyslipidemia as part of comprehensive cardiovascular risk assessment, while the present study provides complementary real‐world evidence on the burden of dyslipidemia in a tertiary‐care outpatient population in Ethiopia.

This study found that age category was an independent predictor of dyslipidemia. Respondents in the 50–64 years’ age group were more than twice as likely to have dyslipidemia compared to those aged 18–34 years. This finding is consistent with studies conducted in high‐income settings like the USA (NHANES) and Germany (DEGS1), as well as local data from Mekelle city, Ethiopia, which demonstrated that lipid abnormalities rise significantly with advancing age [10, 19, 20]. The reason for this increased risk in older adults might be due to physiological changes associated with aging, such as decreased activity of lipoprotein lipase, reduced hepatic LDL receptor expression, and the cumulative effect of long‐term exposure to various metabolic stressors [5, 21].

Occupation status was also significantly associated with dyslipidemia. Specifically, farmers had 62% lower odds of having dyslipidemia compared to unemployed individuals. This finding aligns with the broader body of evidence from the African determinants review, which highlights that occupational physical activity levels are key correlates of lipid profiles [22]. The reduced risk among farmers in this study area might be attributed to the high level of vigorous manual labor and consistent physical activity inherent in farming, which enhances lipid metabolism and maintains higher levels of protective HDL‐C compared to more sedentary or unemployed populations [10, 23].

Regarding BMI category, participants who were obese were four times more likely to have dyslipidemia than those with a normal BMI. This finding is strongly supported by a continental systematic review in Africa and facility‐based cohorts in Ethiopia, which identified obesity as a dominant independent determinant of lipid derangements [22, 24]. This association likely exists because adipose tissue excess leads to an increased flux of free fatty acids to the liver, stimulating the overproduction of VLDL and subsequently raising triglyceride and LDL‐C levels while lowering HDL‐C [22, 25].

The presence of hypertension also significantly predicted the occurrence of dyslipidemia, with hypertensive participants being 81% more likely to have the condition. This result is consistent with the national Ethiopian meta‐analysis and studies among hypertensive adults in Kenya, which show that hypertension and dyslipidemia frequently cluster as part of the metabolic syndrome [5, 13]. The pathophysiological link might be due to shared mechanisms such as insulin resistance and endothelial dysfunction, where elevated blood pressure and abnormal lipids synergistically increase cardiovascular risk [25, 26].

Diabetes mellitus was significantly associated with dyslipidemia in the bivariable analysis but did not remain independently associated with dyslipidemia in the multivariable model. This finding is noteworthy because diabetes is biologically and clinically linked to multiple lipid abnormalities, particularly hypertriglyceridemia, low HDL‐C, and increased atherogenic lipoprotein burden. Evidence from Ethiopia supports a substantial burden of dyslipidemia among people with diabetes; a recent systematic review and meta‐analysis reported a pooled prevalence of diabetic dyslipidemia of 65.7% and identified factors such as physical inactivity and poor glycemic control as important determinants [27]. A previous Ethiopian systematic review and meta‐analysis also demonstrated a high prevalence of hypertriglyceridemia and other lipid abnormalities among people living with diabetes [28]. The absence of an independent association in the present study may therefore reflect differences in the characteristics of the study population and the measurement of diabetes‐related factors. In particular, the present study included a heterogeneous outpatient population rather than a diabetes‐specific cohort, and the analysis did not specifically characterize duration of diabetes, glycemic control, diabetes treatment, or diabetes‐related complications. In addition, adjustment for BMI, hypertension, age, occupation, and other covariates may have attenuated the crude association observed in the bivariable analysis because these factors frequently coexist with diabetes and may share pathways associated with dyslipidemia. The relatively modest sample size may also have limited the ability to detect smaller independent effects. Therefore, the lack of statistical significance should not be interpreted as evidence that diabetes has no relationship with dyslipidemia; rather, it suggests that an independent association was not demonstrated in this particular outpatient population after adjustment for the covariates included in the model.

Several variables that were biologically or epidemiologically plausible determinants of dyslipidemia were not independently associated with the outcome after multivariable adjustment. These included diabetes mellitus, sex, educational level, and residence. The absence of independent associations should be interpreted cautiously because statistical non‐significance does not necessarily indicate the absence of a clinically meaningful relationship. Differences from previous studies may reflect variation in study populations, sample size, study settings, definitions of dyslipidemia, measurement of exposures, and the covariates included in multivariable models. In the present study, the heterogeneous outpatient population may also have reduced differences between demographic subgroups, while adjustment for correlated cardiometabolic factors such as age, BMI, hypertension, and CKD may have attenuated associations observed in crude analyses. In addition, the achieved sample size of 376 participants may have limited the precision of estimates for predictors with smaller effect sizes. These findings therefore emphasize the importance of interpreting both the magnitude and precision of associations, rather than relying exclusively on statistical significance.

### 4.1 Strength of the Study

The study utilized standardized and validated tools to measure the outcome variable (dyslipidemia) and various computable independent variables, ensuring the reliability of the data. A key strength lies in the inclusion of several clinical and metabolic variables, which provided a robust assessment of the factors independently associated with dyslipidemia among adult outpatients. Furthermore, the study incorporated behavioral and lifestyle factors, such as vigorous physical activity and substance use, which are often overlooked in routine clinical settings. The recruitment of participants from the outpatient department of Jimma Medical Center also ensured a diverse representation of both urban and rural dwellers, enhancing the external validity of the findings within the Southwest Ethiopian context.

### 4.2 Limitation of the Study

The cross‐sectional design precludes establishing temporal relationships or causality between the assessed factors and dyslipidemia. Self‐reported behavioral factors, including khat chewing, cigarette smoking, alcohol use, and physical activity, may have been affected by social desirability and recall bias. Although lipid measurements were performed using the same laboratory and analyzer, incomplete information on fasting duration and other pre‐analytical factors may have introduced measurement variability. Consecutive sampling at a single tertiary referral hospital may have introduced selection bias, as referred patients may have a higher burden of chronic disease and cardiovascular risk than the general population, limiting generalizability beyond referral‐level outpatient settings. Exclusion of patients receiving lipid‐lowering therapy may also have introduced selection bias and resulted in an underestimate of the overall burden of dyslipidemia; therefore, the reported prevalence applies specifically to untreated adult outpatients. Finally, residual confounding cannot be excluded because potentially relevant factors, particularly dietary patterns and family history of dyslipidemia or premature cardiovascular disease, were not assessed.

## 5 Conclusion

This study has found a high prevalence of dyslipidemia (60.4%) among adult outpatients attending Jimma Medical Center, highlighting a significant cardiovascular risk burden in the study area. With respect to factors associated with dyslipidemia; older age (50–64 years), obesity (BMI ≥ 30 kg/m2), and the presence of hypertension, were independently found to heighten the risk of developing abnormal lipid profiles. Conversely, occupation as a farmer was found to be a protective factor, significantly reducing the odds of dyslipidemia.

## Author Contributions

**Amare Hailu Ashine:** conceptualization, investigation, methodology, validation, visualization, writing – review and editing, writing – original draft, funding acquisition, supervision. **Besufekad Kero:** conceptualization, investigation, funding acquisition, writing – original draft, writing – review and editing, visualization, validation, methodology, software. **Ermias Habte Gebremichael:** software, methodology, validation, visualization, supervision, writing – review and editing, writing – original draft, funding acquisition, investigation, conceptualization. **Melat Dadigeba:** supervision, methodology, validation, visualization, writing – review and editing, investigation, funding acquisition, writing – original draft, conceptualization. **Yonas Degelo Geremamo:** conceptualization, investigation, funding acquisition, writing – original draft, writing – review and editing, visualization, validation, methodology. **Esmael Mohamed Dawud:** conceptualization, methodology, validation, investigation, funding acquisition, writing – original draft, writing – review and editing, visualization. **Endalew Dessie Birara:** methodology, conceptualization, validation, investigation, visualization, writing – review and editing, writing – original draft, funding acquisition. **Yonas Damtew Bejiga:** conceptualization, methodology, validation, investigation, funding acquisition, visualization, writing – review and editing, writing – original draft. **Sisay Tagese Tafese:** conceptualization, methodology, investigation, validation, funding acquisition, visualization, writing – review and editing, writing – original draft. **Tamirat Godebo Woyimo:** conceptualization, methodology, validation, investigation, funding acquisition, writing – original draft, writing – review and editing, visualization, supervision, software. all authors read and approved the final manuscript and agreed to be accountable for all aspects of the work.

## Funding

No funding was received for this study or manuscript. No external funding source had any role in the study design; collection, analysis, or interpretation of data; writing of the report; or the decision to submit the manuscript for publication.

## Ethics Statement

Ethical clearance was obtained from the Institutional Review Board (IRB) of the Jimma University.

## Consent

Patient confidentiality was strictly maintained throughout the study, with only the principal investigator, supervisor, and data collectors having access to patient information. All data were kept confidential and used solely for research purposes. This study was conducted in accordance with the guidelines of Good Clinical Practice and the Principles of the Declaration of Helsinki. Written informed consent was obtained from all participants prior to their enrollment in the study.

## Conflicts of Interest

The authors declare no conflicts of interest.

## Guarantor Statement

All authors have read and approved the final version of the manuscript. Tamirat Godebo Woyimo, the corresponding author, had full access to all of the data in this study and takes complete responsibility for the integrity of the data and the accuracy of the data analysis.

## Transparency Statement

Tamirat Godebo Woyimo, the corresponding author, affirms that this manuscript is an honest, accurate, and transparent account of the study being reported; that no important aspects of the study have been omitted; and that any discrepancies from the study as planned (and, if relevant, registered) have been explained.

## Data Availability

The data that support the findings of this study are available on request from the corresponding author. The data are not publicly available due to privacy or ethical restrictions.
